# Effects of Mg^2+^ and ATP on YOYO-1 labeling of genomic DNA in single molecule experiments

**DOI:** 10.1016/j.bbrep.2025.102248

**Published:** 2025-09-10

**Authors:** Carl Möller, Dennis Winter, Radhika Nambannor Kunnath, Sriram KK, Fredrik Westerlund

**Affiliations:** Department of Life Sciences, Chalmers University of Technology, Gothenburg, SE, 412 96, Sweden

**Keywords:** Single molecule analysis, Nanofluidic channels, YOYO-1, DNA, ATP, Mg^2+^

## Abstract

Nanofluidic channels have emerged as a suitable tool to study DNA-protein interactions. Many DNA-interacting proteins require ATP to fully function and use Mg^2+^ as a cofactor. Mg^2+^ and ATP are however also known to influence the binding of dyes, such as the commonly used YOYO-1, to DNA. This study investigates the effects of Mg^2+^ ions and ATP on YOYO-1 labeled genomic DNA and shows, via single molecule experiments in nanochannels, that Mg^2+^ reduces the fluorescence intensity of YOYO-1 labeled DNA, as well as the extension of the DNA, at both low and high dye loadings. When combined, ATP counteracts the loss of fluorescence caused by Mg^2+^, but only at comparable concentrations. Additionally, while increasing the photobleaching rate, Mg^2+^ delays dye-mediated photolytic DNA damage, reducing DNA fragmentation in the nanofluidic channels. Determination of the apparent binding constant by bulk measurements corroborates the single molecule observations, suggesting that Mg^2+^ causes dissociation of YOYO-1 from DNA. These findings demonstrate that the addition of Mg^2+^ and ATP poses challenges in DNA-protein studies using nanofluidics, which can be mitigated by optimizing experimental conditions.

## Highlights

• The binding affinity of YOYO-1 fluorescence intensity is influenced by both Mg^2+^ and ATP.
• The binding affinity of YOYO-1 is impacted by both Mg^2+^ and ATP.
• Mg^2+^ reduces and delays photoinduced damage.
• Nanofluidic experiments highlight the need to optimize conditions in single molecule studies of DNA-protein interactions.
• Parallellized nanofluidic chips provide an efficient method to collect and analyze data from large sample sets

## 1 Introduction

Single molecule techniques are important for investigating DNA-protein interactions as they can reveal protein characteristics that are hidden in bulk experiments. Nanofluidic channels, where DNA molecules are stretched by confinement without anchoring, have emerged as a powerful tool to study DNA-protein interactions on the single DNA molecule level [1,2]. Several recent studies have successfully used nanochannels to investigate interactions between genomic DNA and proteins such as the MRE11-RAD50-NBS1 complex [3], CtIP [4], T4 ligase [5], and Hfq [6].

YOYO-1 is the typical dye used for visualization of DNA in nanochannels by fluorescence microscopy. YOYO-1 is non-fluorescent in solution, whereas it shows a ∼400–1000-fold increase in emission quantum yield when bound to double-stranded DNA (dsDNA) [7]. YOYO-1 primarily binds to DNA by bis-intercalation at basepair (bp):dye ratios below 6:1, with an increasing contribution from external binding at higher dye loading [8]. One YOYO-1 molecule occupies ∼3–4 bp [9,10] and the intercalation of YOYO-1 affects both the mechanical and structural properties of DNA [9,[11], [12], [13], [14]]. It has been demonstrated that the contour length of DNA increases linearly with YOYO-1 dye loading reaching a maximum of 38 % increase at dye saturation [9]. The affinity of YOYO-1 to DNA is influenced by the ionic strength [8,15,16] and an ionic strength below 100 mM is preferable for stable YOYO-1 binding [12]. The ionic strength also influences nanoconfined DNA, leading to decreasing extension with increasing ionic strength [17]. These effects make the choice of buffer an integral part of experiments that involve YOYO-1-labeled DNA in nanoconfinement.

YOYO-1 generates single strand breaks (SSBs) on DNA when exposed to light [18]. Two SSBs occurring on opposite strands in close enough proximity result in a double strand break (DSB), which leads to DNA fragmentation. This can be mitigated by removal of oxygen radicals, either by degassing the buffer solution or by adding reducing agents, such as β-mercaptoethanol (BME) or 1,4-dithiothreitol (DTT), or scavengers like the glucose oxidase/catalase system [19].

Metal ions are common cofactors for proteins binding to DNA and Mg^2+^ is one of the most frequent, listed as a cofactor for over 600 enzymes [20,21]. Specifically, Mg^2+^ ions act as cofactors in ATP driven reactions, such as DNA polymerases that synthesize new DNA strands, DNA mismatch repair proteins [22] and DNA ligases that facilitate the joining of DNA strands [23]. ATP must bind Mg^2+^ ions, which stabilize the negative charges of ATP, to become biologically active [24]. This facilitates the hydrolysis of ATP, providing the necessary energy to perform the protein function. The Mg/ATP-complex has been shown to predominantly exist as [MgATP]^2-^ when the Mg^2+^ concentration is within the range of 1–10 mM and the ATP concentration within 0.1–10 mM [25]. Divalent ions like Mg^2+^ also strongly interact with DNA, mainly via electrostatic interactions with the negative charges on the phosphate backbone and has been shown to stabilize the DNA [26].

To our knowledge, no previous study has characterized the effect of Mg^2+^ and ATP on the fluorescence intensity of DNA-bound YOYO-1, either independently or in combination. A previous nanofluidic study briefly mentioned that YOYO-1 is not suitable at Mg^2+^ concentrations of 5 mM or higher [27]. Roushan et al. demonstrated that the extension of YOYO-1-labeled DNA is affected by ATP, with a ∼11 % reduction of DNA extension in the presence of 1 mM ATP [28]. Thus, to ensure the validity of results in experiments involving nanoconfined DNA-protein interactions with physiological concentrations of Mg^2+^ and ATP [29], it is important to characterize the effect of these factors on the fluorescence of YOYO-1-bound DNA.

In this study we have investigated how Mg^2+^, ATP and YOYO-1 influence the fluorescence emission intensity of YOYO-1-labeled and confined DNA. To efficiently collect single molecule data at a vast number of conditions, a recently developed parallelized nanofluidic device was used [30,31]. We observed that the prescence of Mg^2+^ and ATP to DNA-bound YOYO-1 impacts the fluorescence intensity. Additionally, we found a significant difference in photoinduced damage between conditions, where the presence of Mg^2+^ reduced the number of fragmented molecules significantly. By determining the binding constant for all considered conditions we found that the affinity of YOYO-1 for DNA was reduced in the presence of Mg^2+^ and ATP alone, but less so when both were present. For studies focusing on understanding DNA-protein interactions in nanochannel setups, this work establishes the effects of Mg^2+^ and ATP on YOYO-1 labeling of genomic DNA and serves as a guideline for studying ATP-dependent processes on single DNA molecules in nanofluidic channels.

## 2 Materials and methods

### 2.1 Nanofluidic device

A multiplexed nanofluidic device with ten nanochannel modules was used. The device was fabricated in silicon with a 2 μm thick thermal oxide, as described elsewhere [1]. Fabrication of nanochannels was done with e-beam lithography while microchannels were formed with standard photolithography. Inlet and outlet holes were made with deep-reactive-ion etching and micro-nanoconfinement was obtained through wafer scale fusion bonding. The wafer was finally diced into individual chips. Each nanochannel module has 400 nanochannels (100 nm × 150 nm x 420 μm (width x height x length)) and is fed by individual inlets but share an outlet microchannel. A detailed description of the nanofluidic device fabrication can be found in the Supplementary material. The devices were mounted on a custom-made chuck with Luer connectors, enabling easy sample loading and application of pressure-driven flow during experiments.

### 2.2 Single molecule experiments

Samples were prepared as 50 μL reactions with 65 ng [2 μM bp] λ-DNA (ThermoScientific SD0011); 10 mM TRIS-HCL pH 7.5 (Invitrogen 15567-027); 100 mM NaCl; 0.2 or 0.66 μM YOYO-1 (Invitrogen Y3601); 0–10 mM MgCl_2_, and 0–2 mM ATP (Thermo Scientific L14522). The buffer had an ionic strength ranging from 108 to 139 mM. All stocks and reactions were diluted in DEPC water (Fisher BioReagents BP561-1). DNA and YOYO-1 were incubated together at 50 °C for at least 2 h [15].

In the nanofluidic device, DNA molecules were transported hydrodynamically from the sample reservoirs by pressurized N_2_. To gather molecules at the nanochannel entrance, an equal counter pressure was applied to the buffer reservoir. When enough molecules were gathered, a high-pressure pulse pushed the DNA molecules into the nanochannels, the pressure was switched off and the molecules were imaged.

Imaging was done using a fluorescence microscope (Zeiss AxioObserver.Z1) equipped with a 100 × (1× optovar) oil immersion objective (NA = 1.46, Zeiss) and a Photometrics prime 95B camera. For excitation, a Zeiss Colibri 7 LED was used, set to 469 nm and coupled with Filter Set 44 FITC (Zeiss). For experiments to determine fluorescence intensity and extension, images were captured with the following settings: 10 % light intensity, 15 frames, 100 ms exposure. For the photo damage experiments, images were captured at 50 % light intensity, 50 ms exposure, and at least 30 s of imaging.

### 2.3 Data analysis

All data analysis was performed using custom-written MATLAB or R-based scripts. As described previously [3,31], the custom MATLAB script first detects the DNA molecules in an image stack and then generates kymographs for each individual molecule. The extension, the corresponding standard deviation, and the emission intensity for each DNA molecule were then extracted from the corresponding kymograph [32].

### 2.4 Photolysis and gel electrophoresis

Samples were prepared with 16.25 ng linearized pAAV-U6-sgRNA-CMV-GFP vector (Addgene # 85451); 10 mM TRIS-HCL pH 7.5 (Invitrogen 15567-027); 100 mM NaCl; 0.1 μM YOYO-1 (Invitrogen Y3601) and 0–5 mM MgCl_2_. All stocks and reactions were diluted in DEPC water (Fisher BioReagents BP561-1) and incubated for 2 h at 50 °C before being distributed in 25 μL aliquots across a 96-well Half Area Black/Clear Flat Bottom plate (Corning 3881). The samples were exposed to 475 nm light from a Zeiss Colibri 7 via a 5x (NA = 0.13) objective. The intensity was measured with a ThorLabs PM30 optical power meter to 100 mW, equating to a power density of 0.52 W/cm^2^. The samples were subsequently analyzed on a 0.8 % agarose (1xTAE) gel at 8 V/cm for 2 h.

### 2.5 Fluorescence measurements

Bulk fluorescence measurements were performed on a FluoStar Optima microplate reader (BMG Labtech) in 96-well Half Area Black/Clear Flat Bottom plates (Corning 3881). Each condition was set up as a 50 μL reaction with 32.5 ng [1 μM bp] λ-DNA (Thermo Scientific SD0011); 10 mM TRIS-HCL pH 7.5 (Invitrogen 15567-027); 100 mM NaCl; 0–1 μM YOYO-1 (Invitrogen Y3601); 0–10 mM MgCl_2_ and 0–2 mM ATP (Thermo Scientific L14522). All stocks and reactions were diluted in DEPC water (Fisher BioReagents BP561-1).

## 3 Results and discussion

**Fig. 1:**
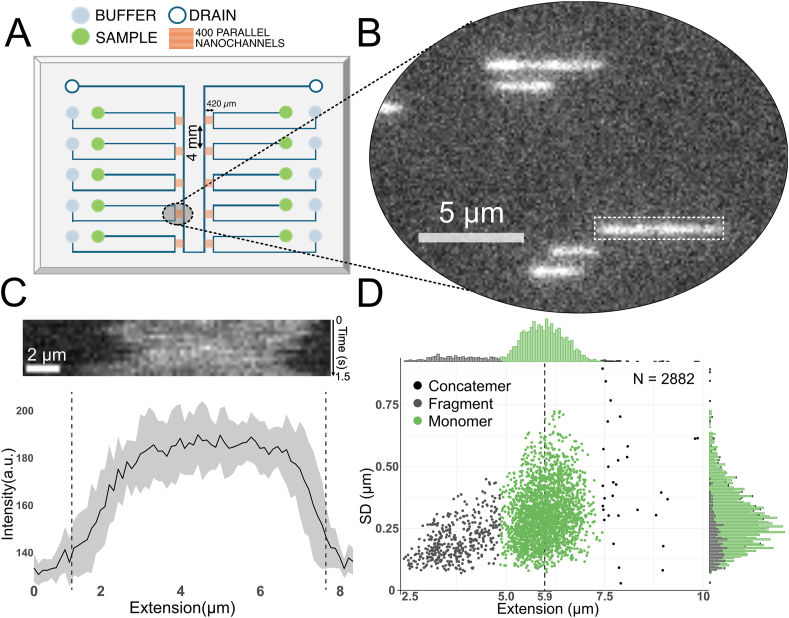
**A.** Schematic of the nanofluidic device capable of processing 10 samples. Pressure is applied to the sample well (green) to flow the DNA through the microchannels towards the nanochannels entrance. As the DNA gathers at the nanochannel entrance, pressure is simultaneously applied to the sample (green) and buffer reservoirs (blue), directing all flow through the nanochannel array. This forces the DNA to enter the nanochannels, where it is stretched and imaged. **B.** Image showing a partial field of view from a nanofluidic array with YOYO-1-stained λ-DNA. **C.** Top shows the kymograph generated from time dependent imaging of the nanochannel stretched DNA molecule outlined by the dashed box in **B**. Below is a time-averaged intensity profile for a representative monomeric λ-DNA stained at a 1:10 dye:bp ratio. The vertical lines indicate the edges of the molecule detected in the box (white, with dashed lines) in the image. **D.** Scatterplot from a typical control sample (λ-DNA stained at 1:10 dye:bp) with the extension for each molecule plotted against the standard deviation of its extension (SD). The marginal histogram visualizes the distribution along each axis.

This study investigates the influence of Mg^2+^ and ATP on YOYO-1 labeling of DNA, focusing on single molecule experiments using nanofluidic channels. We defined a background buffer constituted by 10 mM TRIS and 100 mM NaCl at pH 7.5. The buffer conditions were designed to mimic physiological ionic strength, which typically ranges between 100 and 200 mM [34], since most biochemical reactions are expected to be investigated within this range. The experimental range for Mg^2+^ (0–5 mM) and ATP (0–2 mM) was chosen to reflect typical biochemical reactions. To enable efficient investigations at varying concentrations of Mg^2+^, ATP and YOYO-1, a multiplexed and automated nanofluidic chip with ten nanochannel modules was used [30] (Fig. 1A). The device was operated via an automated pressure controller (OB1 MK3, Elveflow) and an automated imaging routine on the microscope so that data for thousands of DNA molecules at multiple experimental conditions could be collected in a parallel and automated fashion. In short, the λ-DNA molecules were transported from the sample wells through the microchannels by application of pressurized N_2_, creating a hydrodynamic flow. By applying an equal counter pressure to the buffer wells the molecules are forced towards the nanochannel entrance. By adjusting the pressure to a sufficiently low level the molecules gather at the nanochannel entrance, but do not enter. After collecting a sufficient number of molecules at the nanochannel entrance they are pushed in by a high-pressure pulse (from both the sample and buffer wells); this applies enough force for the molecules to overcome the entropic barrier and enter the nanochannels. Imaging is performed while the pressure is turned off. After imaging, a second high-pressure pulse expels the molecules from the nanochannels towards the drain and the cycle repeats. Fig. 1B shows a representative image of a DNA molecule labeled with YOYO-1 dye stretched in a nanochannel. The stretched molecules were imaged over time, generating a short movie. To quantify the intensity and extension, each molecule was segmented (Fig. 1B, dashed box) and each time frame stacked to generate a kymograph (Fig. 1C, top). The emission intensity along the molecule (Fig. 1C, bottom, solid black line) and extension (Fig. 1C, bottom, dashed vertical lines) for each DNA molecule is then determined by taking the average along the time and distance axis, respectively. The λ-DNA (48.5 kbp) used has a 12-base single-stranded overhang that can either self-hybridize to form circular DNA or hybridize to the overhang of another DNA molecule to form concatemers. Fig. 1D shows a scatterplot from a typical λ-DNA sample without any Mg^2+^ or ATP present. The plot visualizes the extension of each molecule plotted against the standard deviation of its extension. Under confinement, thermal energy will give rise to fluctuations of the molecule extension, which is quantified by the standard deviation of the extension. From the extension and standard deviation, it is possible to categorize the molecules based on their conformations [3,4,31]. In the data reported in Fig. 1D, a majority of the molecules belong to one cluster that represents monomeric linear λ-DNA, whereas fragmented and circular molecules have a lower extension and/or standard deviation [33]. To ensure comparability between datasets, only molecules categorized as full-length linear monomers were included in the analysis below.

### 3.1 Mg^2+^-ions and ATP influence YOYO-1 fluorescence intensity in single molecule experiments

**Fig. 2:**
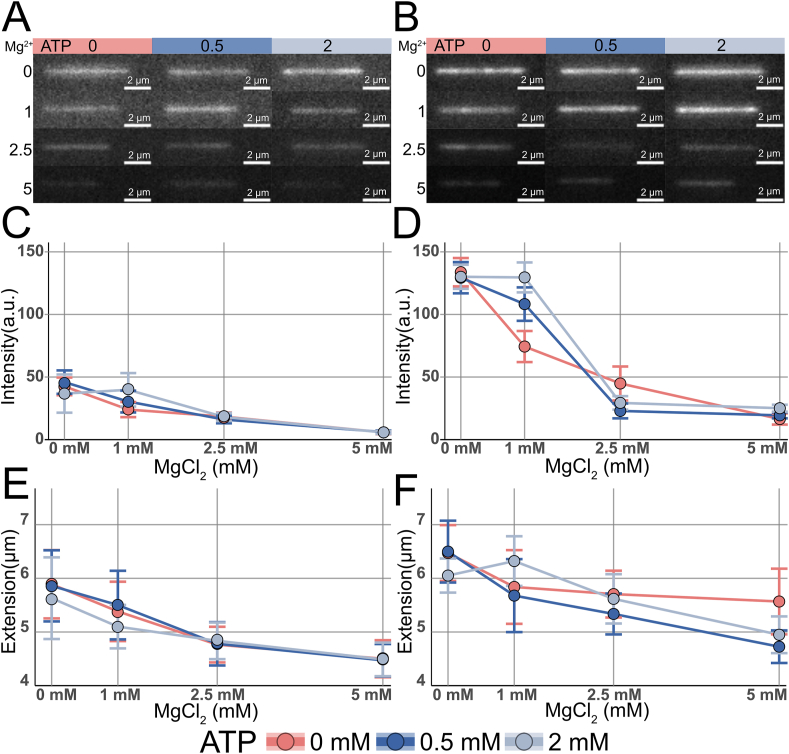
Representative microscope images of monomeric λ-DNA confined in nanochannels at varying ATP and MgCl_2_ concentrations at a 1:10 (**A**) and 1:3 (**B**) dye:bp ratio. Each individual image has a scalebar indicating 2 μm and each set has a calibration bar showing the intensity range (a.u.) Mean, background adjusted, fluorescence intensity of monomeric λ-DNA at varying ATP and Mg^2+^ concentrations at a 1:10 (**C**) and 1:3 (**D**) dye:bp ratio, respectively. Average DNA extension with varying ATP and Mg^2+^ concentrations at a 1:10 (**E**) and 1:3 (**F**) dye:bp ratio. Color legend applies to all subfigures.

A 1:10 dye:bp ratio is common for nanofluidic studies of DNA-protein interactions, and the primary binding mode of YOYO-1 to DNA at low dye concentrations is bis-intercalation. The 1:3 dye:bp ratio was included to investigate the effect of Mg^2+^ and ATP at a higher dye loading, where YOYO-1 is also bound externally on the DNA [35]. Fig. 2A–B shows representative images from single molecule experiments with λ-DNA stained with YOYO-1 at either 1:10 (Fig. 2A) or 1:3 (Fig. 2B) dye:bp ratio and varying Mg^2+^ and ATP concentrations. From a qualitative assessment of the image data there is a definite decrease in intensity along the Mg^2+^ gradient as well as a decrease in extension for both dye:bp ratios. Fig. 2C–D report the quantitation from a large set of molecules for each condition. The data confirms the previous observations for both dye:bp ratios, the intensity decreases significantly with increasing Mg^2+^ concentration. However, comparing the intensity levels between 0 and 1 mM Mg^2+^, the addition of ATP seemingly mitigates this effect. At the lowest Mg^2+^ concentration (1 mM) and the highest ATP concentration (2 mM), the observed intensity is comparable to control, which is due to the formation of the MgATP complex which removes free Mg^2+^ ions from solution. Importantly, the intensity drop with increasing Mg^2+^ concentration is similar for both dye:bp ratios. However, at higher Mg^2+^ concentrations, where there is an excess of Mg^2+^, the effect of ATP is not significant as there is excess Mg^2+^ in the solution that is not forming the MgATP complex. Hence, this suggests that ATP and Mg^2+^ should be at comparable concentrations to prevent the loss of YOYO-1 fluorescence in the presence of Mg^2+^.

Mg^2+^ and ATP also affect DNA extension in nanochannels. Fig. 2C–D report the average DNA extension for both dye:bp ratios. The overall trend is a decrease in extension with increasing Mg^2+^ concentration. ATP does not alter this effect for the lower dye:bp ratio, whereas a small deviation is observed for the higher dye loading. The observed results are expected and in accordance with previous results [30] obtained using the same multiplexed nanofluidic chip, indicating that the extension of DNA is dependent on the ionic strength [17]. However, from established models describing DNA behavior in confinement [1,[36], [37], [38]], it is possible to extrapolate a theoretical extension (Supplementary Figure 1) that indicates the expected average extension of a λ-DNA molecule under a given dye:bp ratio, ionic strength and confinement. By taking the ratio of the observed and theoretical extensions (Ext_O_/Ext_T_), it is possible to identify whether there are additional effects, in addition to the increased ionic strength, causing the change in extension. If the extension would change solely due to the increase in ionic strength, the ratio would be ∼1 for all tested conditions. However, for our experiments, the Ext_O_/Ext_T_ is below one (<1) for all conditions (above 1 mM Mg^2+^) and decreases with increasing Mg^2+^ concentration (Supplementary Figure 2). This could be interpreted as evidence for competitive binding between Mg^2+^ and YOYO-1 where magnesium hinders the YOYO-1 intercalation with the DNA, thus reducing the extension of the molecule and the observed fluorescence intensity. Additionally, this could also be an indication of Mg^2+^ induced compaction of the DNA [39]. Mg^2+^ binds electrostatically to DNA, thereby reducing the repulsion between the two DNA strands, which leads to compaction of DNA [40]. The compaction of DNA alters the distance between YOYO-1 molecules, which could lead to YOYO-1 self-quenching [41], which would, in turn, potentially serve as an additional explanation to the observed decline in fluorescence intensity.

### 3.2 Mg^2+^ delays photoinduced DNA damage

**Fig. 3:**
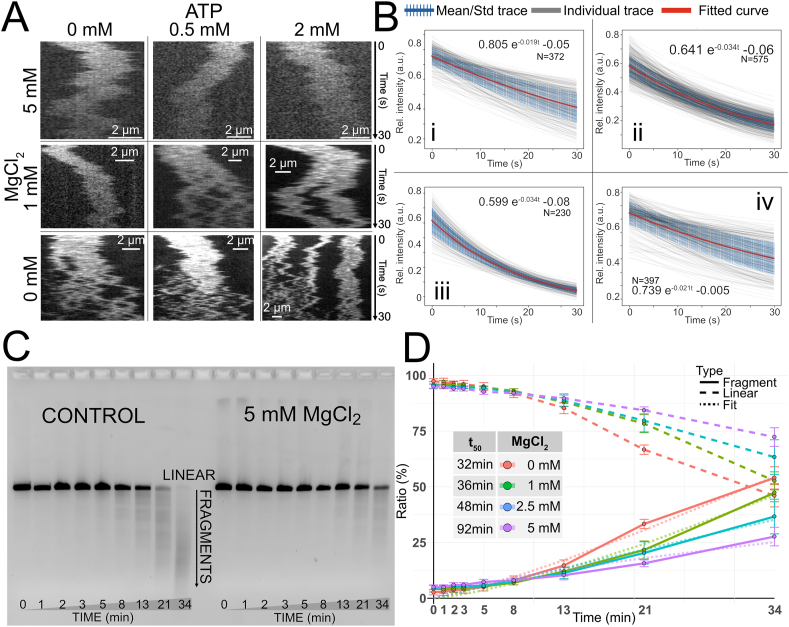
**A.** Representative kymographs from bleaching/fragmentation experiments with λ-DNA stained with YOYO-1 at a 1:10 bp:dye ratio and varying concentrations of Mg^2+^ and ATP. **B.** Individual photobleaching traces (gray lines) from a large set of molecules with the mean trace and standard deviation (blue with error bars) for 0 mM (i), 1 mM (ii) and 5 mM (iii) Mg^2+^ and 1 mM Mg^2+^ + 2 mM ATP (iv) with fitted curves (${Ae}^{-\alpha \tau }+B$), orange lines. **C.** Gel electrophoresis of photobleached DNA with and without Mg^2+^. **D.** Densiometric quantification of results from gel electrophoresis. The fragmented DNA was defined as all signal below the linear band. The dashed line is the normalized intensity of the intact linear fraction, and the solid line is the fragment fraction. The dotted line is the fitted power-law ($y=rt^{n}$) describing the fragmentation rate for each condition. The table reports the extrapolated time at which 50 % of the DNA is fragmented (t_50_).

The movies for each DNA molecule were turned into kymographs. When inspecting the kymographs, two important trends were identified. First, the YOYO-1 dye bleaches when exposed to light, seen as a gradual decrease in emission intensity, and secondly, the DNA starts to fragment. Most importantly, both effects varied with Mg^2+^ and ATP concentration (Fig. 3A). To further characterize the influence of Mg^2+^ on photobleaching, data was collected at 1:10 dye:bp by continuous imaging until the signal was non-detectable. Fig. 3B reports the intensity traces for several hundreds of molecules, and a decay curve (A $e^{-\alpha \tau }+B$) fitted to the average of all traces, for samples at four different conditions. By comparing the rate constant (α), it is apparent that the intensity decays faster in the presence of Mg^2+^. However, with the addition of ATP, the decay rate is comparable to the rate in the control sample.

The second effect observed was a delayed onset of DNA fragmentation with increasing Mg^2+^ concentration (Fig. 3A). Addition of ATP (0.5 mM and 2 mM) increased fragmentation, demonstrating that Mg^2+^-ATP interactions counteract the effects of Mg^2+^alone. At 5 mM Mg^2+^, we did not observe DNA fragmentation, even at 2 mM ATP. To further verify and quantify the DNA fragmentation observed in the single molecule experiments, we performed a bulk assay with YOYO-1-stained DNA at varying concentrations of Mg^2+^. Each sample was exposed to light in a 96-well clear bottom plate using the fluorescence microscope. After exposure, the samples were analyzed with gel electrophoresis and quantified using densiometric analysis by comparing the intensity of the band of intact linear DNA and the smear beneath it, which contained the fragmented DNA (Fig. 3C). The quantification in Fig. 3D shows that the fragmentation was significantly faster in the control sample compared to the sample with 5 mM Mg^2+^. By fitting a curve ($y=rt^{n}$) to the data, it is possible to extrapolate to the time at which 50 % of the DNA is fragmented (t_50_). t_50_ increases drastically with increasing Mg^2+^ concentration. The explanation behind this is most likely a decreased amount of YOYO-1 bound at higher Mg^2+^. A second, contributing explanation could be the stabilization of DNA by Mg^2+^. Since fragmentation from photolytic damage happens when nicks on opposite strands occur sufficiently close, an increased stabilization decreases this distance, thus increasing the time until two nicks occur close enough.

### 3.3 Mg^2+^- and ATP dependent binding affinity of YOYO-1

**Fig. 4:**
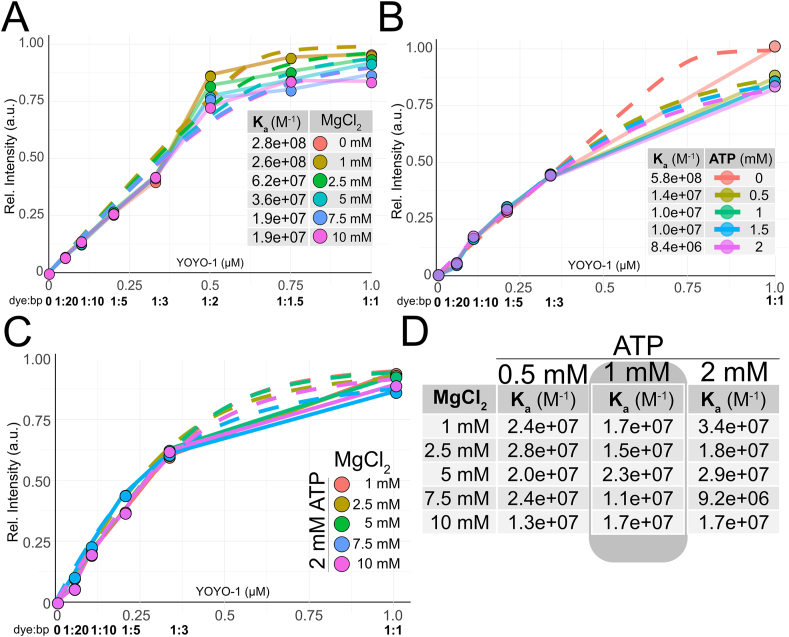
**A.** Normalized fluorescence at increasing YOYO-1 concentrations at a constant DNA concentration of 1 μM (bp) and increasing concentrations of Mg^2+^. Dashed lines represent the fitted MvH model, and the table inset reports the K_a_ values. **B.** Normalized fluorescence at increasing YOYO-1 concentrations at a constant DNA concentration of 1 μM (bp) and increasing concentrations of ATP. Dashed lines represent the fitted MvH model, and the table inset reports the K_a_ values. **C.** Normalized fluorescence at increasing YOYO-1 concentrations at a constant DNA concentration of 1 μM (bp), constant ATP concentration of 2 mM and increasing concentrations of Mg^2+^ Dashed lines represent the fitted MvH model. **D**. Table with the associated constants from experiments at three fixed ATP concentrations and varying Mg^2+^concentrations.

The addition of Mg^2+^ to YOYO-1-labeled DNA causes a decrease in emission intensity, decreased extension, increased photobleaching, and a decrease in photoinduced fragmentation. One contributing explanation could be a decrease in YOYO-1 binding to DNA in the presence of Mg^2+^. To confirm this, we performed bulk experiments to determine the apparent binding constant of YOYO-1 to DNA. First, the influence of Mg^2+^ and ATP was investigated separately. When fitting binding curves, it is important that the maximum fluorescence intensity is achieved. To ensure this, several “supersaturated” YOYO-1 levels were included in an initial iteration (Supplementary Figure 3). These experiments confirmed that the emission intensity levelled out at a dye:bp ratio of 1:1. Fig. 4A shows the normalized intensity for YOYO-1:DNA ratios between 1:20 and 1:1 at several Mg^2+^ concentrations. Binding constants (K_a_) and binding site sizes (n) were extracted by fitting a simplified McGhee von Hippel (MvH) binding model:

(1) $$\left[L_{T}\right]=\left[L_{f}\right]+\frac{K_{a}\left[L_{f}\right]}{1+K_{a}\left[L_{f}\right]}\left(\frac{\left[S_{T}\right]}{n}\right)$$

where L_T_ and L_f_ are the total and free concentration of ligand, S_T_ is the concentration in basepairs, K_a_ is the apparent binding constant and n is the size of the binding site (see Supplementary material for a full derivation). K_a_ decreases from 2.8x10^8^ M^-1^ to 1.9x10^7^ M^-1^ with increasing Mg^2+^, in agreement with previous observations from force extension experiments [12], confirming that Mg^2+^ decreases the binding affinity of YOYO-1. Supplementary Tables 1 and 2 show the extracted binding constants and n from fitting of the described model along with the goodness of fit metrics. Similar analyses using a Scatchard analysis and a Hill plot, supporting the analysis, are shown in Supplementary Fig. 4A–B.

Next, we determined K_a_ at increasing concentrations of ATP (Fig. 4B) without Mg^2+^ present. Only at a dye:bp ratio of 1:1 did the addition of ATP decrease the YOYO-1 emission (∼20 %). K_a_ was extracted for each condition using the same MvH model. K_a_ is comparable for the control samples in both experimental series (Fig. 4A–B) and, similar to the findings for Mg^2+^, decreases with increasing ATP concentration, with the largest difference between 0 and 0.5 mM ATP. It should be noted that ATP could have a gradual effect at lower concentrations than investigated here and that we therefore only observe the plateau reached after saturation. Supporting results from Hill plot analysis are shown in Supplementary Fig. 5A–B.

Finally, experiments were performed at varying Mg^2+^ concentrations at fixed levels of ATP. Fig. 4C shows the normalized intensity for YOYO-1:DNA ratios between 1:20 to 1:1 for each Mg^2+^ concentration at 2 mM ATP. The data follows the observations in Fig. 4A–B with the slight difference that there is no real intensity drop at 1:1 dye:bp, as seen with Mg^2+^ and ATP alone, indicating that the complexation of Mg^2+^ and ATP indeed influences YOYO-1 binding to DNA. Binding constants were extracted for each condition using the same MvH model (Fig. 4D). The binding site size n was found to be around 2, in line with previous reports [42], for most conditions. Notably, the effects are in general smaller when ATP and Mg^2+^ are added together than for the two components alone. Supporting results from Hill plot analysis are shown in Supplementary Fig. 5C–D.

## 4 Conclusion

To conclude, our findings demonstrate that Mg^2+^ significantly influences the emission intensity from DNA-bound YOYO-1, while ATP only has minor effects on its own. By comparing the observed and theoretical extensions, it was found that the addition of Mg^2+^ decreases the extension beyond what would be expected from the increased ionic strength. This could be explained by a loss of bound YOYO-1 since intercalation by YOYO-1 contributes to the contour length of the DNA. The influence of Mg^2+^ and ATP on YOYO-1 binding was confirmed by determination of the apparent binding constants. When combined, ATP decreases the effect of Mg^2+^ on the fluorescence of DNA bound YOYO-1, highlighting the importance of considering them as a complex rather than as individual ions. The fact that ATP counteracts the effect of Mg^2+^ is beneficial, since it allows studies of DNA-protein interactions at higher Mg^2+^ concentrations than for Mg^2+^ alone. Within the physiological range of concentrations, an excess of ATP (2 mM) in the presence of 1 mM Mg^2+^ leads to the formation of the [MgATP]^2-^ complex, which removes free Mg^2+^ ions in the solution, thus counteracting the loss of fluorescence in DNA bound YOYO-1 caused by Mg^2+^ alone. Additionally, Mg^2+^ reduces photoinduced DNA fragmentation, which can be explained by the lowered binding constants. Interestingly, complementary bulk experiments did not identify all the nuances from the single molecule experiments, highlighting the importance of optimizing experimental conditions for nanofluidics at the single molecule level. Our results serve as a guideline for concentrations and conditions to use in future studies of DNA-protein dynamics in nanofluidic channels when Mg^2+^, ATP, and YOYO-1 are present.

## CRediT authorship contribution statement

**Carl Möller:** Writing – original draft, Visualization, Resources, Methodology, Investigation, Formal analysis, Data curation, Conceptualization. **Dennis Winter:** Writing – original draft, Resources, Methodology, Investigation, Data curation. **Radhika Nambannor Kunnath:** Writing – review & editing, Resources, Methodology, Investigation. **Sriram KK:** Writing – original draft, Resources, Investigation. **Fredrik Westerlund:** Writing – original draft, Supervision, Project administration, Funding acquisition, Conceptualization.

## and code availability

The imaging data and analysis routines are available and can be obtained by making a reasonable request to the corresponding author.

## Declaration of generative AI and AI-assisted technologies in the writing process

This document was created with the assistance of generative AI and AI-assisted technologies. Specifically, the following tools and services were utilized:

**Microsoft Copilot**: Used to summarize text based on user input and provided documents. **DeepSeek**: Employed for optimization of binding model implementations.

## Funding

This project was funded by the 10.13039/501100000781European Research Council in the form of an ERC consolidator grants (no. 866238), the European Union under the RepState Project (Marie Skłodowska-Curie Grant Agreement No. 101073485) and the 10.13039/501100004359Swedish Research Council (no. 2020–03400).

## Declaration of competing interest

The authors declare that they have no known competing financial interests or personal relationships that could have appeared to influence the work reported in this paper.

## Data Availability

Data will be made available on request.

## References

[1] Frykholm K., Müller V., Dorfman K.D., Westerlund F. (2022). DNA in nanochannels: theory and applications. Q. Rev. Biophys..

[2] Frykholm K., Nyberg L.K., Westerlund F. (2017). Exploring DNA-protein interactions on the single DNA molecule level using nanofluidic tools. Integr. Biol..

[3] Möller C. (2024). Xrs2/NBS1 promote end-bridging activity of the MRE11-RAD50 complex. Biochem. Biophys. Res. Commun..

[4] Öz R. (2020). Phosphorylated CtIP bridges DNA to promote annealing of broken ends. Proc. Natl. Acad. Sci. U. S. A..

[5] Roushan M. (2014). Probing transient protein-mediated DNA linkages using nanoconfinement. Biomicrofluidics.

[6] Malabirade A. (2017). Compaction and condensation of DNA mediated by the C-terminal domain of Hfq. Nucleic Acids Res..

[7] Rye H.S. (1992). Stable fluorescent complexes of double-stranded DNA with bis-intercalating asymmetric cyanine dyes: properties and applications. Nucleic Acids Res..

[8] Larsson A., Carlsson C., Jonsson M. (1995). Characterization of the binding of YO to [poly(dA‐dT)]2 and [poly(dG‐dC)]2, and of the fluorescent properties of YO and YOYO complexed with the polynucleotides and double‐stranded DNA. Biopolymers.

[9] Kundukad B., Yan J., Doyle P.S. (2014). Effect of YOYO-1 on the mechanical properties of DNA. Soft Matter.

[10] Wang Y., Sischka A., Walhorn V., Tönsing K., Anselmetti D. (2016). Nanomechanics of fluorescent DNA dyes on DNA investigated by magnetic tweezers. Biophys. J..

[11] Murade C.U., Subramaniam V., Otto C., Bennink M.L. (2009). Interaction of oxazole yellow dyes with DNA studied with hybrid optical tweezers and fluorescence microscopy. Biophys. J..

[12] Günther K., Mertig M., Seidel R. (2010). Mechanical and structural properties of YOYO-1 complexed DNA. Nucleic Acids Res..

[13] Tegenfeldt J.O. (2004). The dynamics of genomic-length DNA molecules in 100-nm channels. Proc. Natl. Acad. Sci. U. S. A..

[14] Iarko V. (2015). Extension of nanoconfined DNA: quantitative comparison between experiment and theory. Phys. Rev..

[15] Nyberg L., Persson F., Åkerman B., Westerlund F. (2013). Heterogeneous staining: a tool for studies of how fluorescent dyes affect the physical properties of DNA. Nucleic Acids Res..

[16] Gautam D., Pandey S., Chen J. (2023). Effect of flow rate and ionic strength on the stabilities of YOYO-1 and YO-PRO-1 intercalated in DNA molecules. J. Phys. Chem. B.

[17] Reisner W. (2007). Nanoconfinement-enhanced conformational response of single DNA molecules to changes in ionic environment. Phys. Rev. Lett..

[18] Åkerman B., Tuite E. (1996). Single- and double-strand photocleavage of DNA by YO, YOYO and TOTO. Nucleic Acids Res..

[19] Aitken C.E., Marshall R.A., Puglisi J.D. (2008). An oxygen scavenging system for Improvement of dye stability in single-molecule fluorescence experiments. Biophys. J..

[20] Bairoch A. (2000). The ENZYME database in 2000. Nucleic Acids Res..

[21] Foster A.W., Osman D., Robinson N.J. (2014). Metal preferences and metallation. J. Biol. Chem..

[22] Borsellini A., Kunetsky V., Friedhoff P., Lamers M.H. (2022). Cryogenic electron microscopy structures reveal how ATP and DNA binding in MutS coordinates sequential steps of DNA mismatch repair. Nat. Struct. Mol. Biol..

[23] Doherty A.J., Suh S.W. (2000). Structural and mechanistic conservation in DNA ligases. Nucleic Acids Res..

[24] Pilotelle-Bunner A., Cornelius F., Sebban P., Kuchel P.W., Clarke R.J. (2009). Mechanism of Mg2+ binding in the Na+,K+-ATPase. Biophys. J..

[25] Storer A.C., Cornish-Bowden A. (1976). Concentration of MgATP2- and other ions in solution. Calculation of the true concentrations of species present in mixtures of associating ions. Biochem. J..

[26] Serec K., Babić S.D., Podgornik R., Tomić S. (2016). Effect of magnesium ions on the structure of DNA thin films: an infrared spectroscopy study. Nucleic Acids Res..

[27] Roushan M. (2018). Motor-like DNA motion due to an ATP-hydrolyzing protein under nanoconfinement. Scientific Reports 2018.

[28] Roushan M., Azad Z., Lim S.F., Wang H., Riehn R. (2015). Interference of ATP with the fluorescent probes YOYO-1 andYOYO-3 modifies the mechanical properties of intercalator-stained DNA confined in nanochannels. Mikrochim. Acta.

[29] Tomita A. (2017). ATP-dependent modulation of MgtE in Mg2+ homeostasis. Nat. Commun..

[30] Kk S. (2021). A parallelized nanofluidic device for high-throughput optical DNA Mapping of bacterial plasmids. Micromachines.

[31] Pavlova E. (2025). High-throughput single-molecule nanofluidic studies on B. subtilis Rok protein interaction with DNA. QRB Discov.

[32] Frykholm K. (2015). Fast size-determination of intact bacterial plasmids using nanofluidic channels. Lab Chip.

[33] Alizadehheidari M. (2015). Nanoconfined circular and linear DNA: equilibrium conformations and Unfolding Kinetics. Macromolecules.

[34] Scott S. (2019). Single-molecule visualization of the effects of ionic strength and crowding on structure-mediated interactions in supercoiled DNA molecules. Nucleic Acids Res..

[35] Larsson A., Carlsson C., Jonsson M., Albinsson B. (1994). Characterization of the binding of the fluorescent dyes YO and YOYO to DNA by polarized light spectroscopy. J. Am. Chem. Soc..

[36] Reisner W., Pedersen J.N., Austin R.H. (2012). DNA confinement in nanochannels: physics and biological applications. Rep. Prog. Phys..

[37] Werner E., Mehlig B. (2014). Confined polymers in the extended de Gennes regime. Phys. Rev..

[38] Gupta D. (2015). Experimental evidence of weak excluded volume effects for nanochannel confined DNA. ACS Macro Lett..

[39] Tongu C. (2016). Divalent cation shrinks DNA but inhibits its compaction with trivalent cation. J. Chem. Phys..

[40] Bloomfield V.A. (1997). DNA condensation by multivalent cations. Biopolymers.

[41] Wang L., Pyle J.R., Cimatu K.L.A., Chen J. (2018). Ultrafast transient absorption spectra of photoexcited YOYO-1 molecules call for additional investigations of their fluorescence quenching mechanism. J. Photochem. Photobiol., A: Chem.

[42] Fernández-Sierra M., Quiñones E. (2015). Assays for the determination of the activity of DNA nucleases based on the fluorometric properties of the YOYO dye. Arch. Biochem. Biophys..

