# Supplementary material for: Effects of Mg2+ and ATP on YOYO-1 labeling of genomic DNA in single molecule experiments
Source: Biochem Biophys Rep. 2025 Sep 10;44:102248. doi: 10.1016/j.bbrep.2025.102248 (PMC12455114; doi:10.1016/j.bbrep.2025.102248)
Supplement: Multimedia component 1 [file mmc1.docx]

Effects of Mg^2+^ and ATP on YOYO-1 labeling of genomic DNA in single molecule experiments

Carl Möller^a*^, Dennis Winter^a*^, Radhika Nambannor Kunnath^a^, Sriram KK^a^, Fredrik Westerlund^a^**

*a. Department of Life Sciences, Chalmers University of Technology, Gothenburg, SE, 412 96, Sweden*

*These authors contributed equally.

**Corresponding author: Fredrik Westerlund, *[fredrik.westerlund@chalmers.se](mailto:fredrik.westerlund@chalmers.se)*

[Nanofluidic device fabrication 1](#_Toc716452227)

[Ionic strength and theoretical extension 2](#_Toc311710644)

[Single molecule clustering 4](#_Toc1603967449)

[Determination of binding constants 4](#_Toc2073587272)

[McGhee von Hippel 4](#_Toc247659939)

[Scatchard plot and Hill plot 5](#_Toc1387412485)

[References 7](#_Toc405720481)

All analysis and visualization was performed in R^1^ with custom functions and ggplot2^2^.

# Nanofluidic device fabrication

A fresh 500 μm thick, n-type 4” Si wafer (Si-mat, Germany) was subjected to standard cleaning process (SC1 and SC2) to remove any organic or metallic contaminants, particles and residues from the wafer surface. Next, a 2 μm thick wet oxide was grown using an oxidation furnace (Centrotherm, 1050 °C, 780 minutes). Next, alignment marks for multi-step micro-nanofabrication were fabricated using photolithography (PL, S1813, Shipley Inc., USA) and etched to 850 nm using reactive ion etching (RIE, 50 sccm Ar, 50 sccm CHF3, 150 W RF-power, 30 mbar pressure). The remaining photoresist after etching was removed using piranha cleaning (con. H_2_SO_4_ and H_2_O_2_ at 2:1 ratio, 120 °C for 10 minutes), the wafer rinsed with DI-water and dried using a nitrogen gun.

For electron beam lithography of nanochannels (EBL, JEOL JBX-9300FS), a 20 nm thick chromium layer was deposited using e-gun evaporation (Lesker PVD 225), followed by sputtering of 24 nm thick SiO_2_ (FHR MS-150 Sputter) to form a hard mask for RIE of nanochannels. Electron beam lithography was carried out using AR-P 6200.13 resist (diluted in Anisole, 1: 1 ratio, Allresist GmBH) and then etched using RIE (50 sccm NF_3_, 25 W RF-power, 8 mbar pressure) to get 100 nm deep nanochannels. Sputtered SiO_2_ was removed with a CF_4_ etch (Plasmatherm RIE, 40 sccm CF4, 100 W RF-power, 100 mT pressure) and the chromium layer was removed using a chromium etchant (SunChem AB, Sweden). The substrate was once again cleaned using a piranha solution. Microchannels were then fabricated using PL and RIE, with the same protocol used for obtaining alignment marks, but to an etch depth of 1.8 μm. The substrate was once again cleaned using piranha solution.

Through hole structures (∅ = 1 mm) were fabricated using PL (AZ4562, MicroChem GmBH) and deep reactive ion etching (DRIE, Oxford Plasma Pro 100), involving Bosch high rate-etching of ~10 µm/min. Fusion bonding of the substrate containing the micro- and nanofluidic features was then carried out. For this, the substrate was first subjected to oxygen plasma (40 sccm O2, 250 W RF-power, 500 mT pressure, 1 minute) and then a piranha cleaned borofloat glass (170 μm thick, double side polished, Si-Mat) was subjected to oxygen plasma (40 sccm O2, 350 W RF-power, 500 mT pressure, 2 minutes). The plasma treated surfaces were brought together and immediately formed a temporary bonding. This pair was then subjected to fusion bonding at 550 °C for 5 hours. The fusion bonding was done by gradually increasing the temperature required for bonding and slowly cooling down afterwards, to avoid any cracking due to different thermal coefficients of Si wafer and borofloat glass. Finally, four individual chips of 27 x 36 mm^2^ were obtained using an automated dicing saw (Disco DAD3350).

# Ionic strength and theoretical extension

The theoretical extension of a DNA molecule in confinement is dependent on the contour length (L), effective width (w), persistence length (l_p_) and the geometry of the nano channel(D)^3,4^ . The scaling of the extension in the de Gennes regime^3^ is described by Equation 1:

$X \cong L\left( \frac{wl_{p}}{D^{2}} \right)^{\frac{1}{3}}$ (1)

At the specific channel dimensions used in this study (100x150 nm), the extension is best described by scaling in the extended de Gennes regime^3^ by adding a factor α= 1.1765 to the scaling equation^5–7^ .

If the nano channel has an asymmetrical geometry, e.g. D1≠D2, D can be replaced by D_av_^4^  according to Equation 2:

$D_{Av}=\left( D_{1}D_{2} \right)^{\frac{1}{2}}$ (2)

The persistence length^3^ can be approximated by Equation 3:

$l_{p}\left[ nm \right]=\left( 23+33\gamma+26\gamma^{2} \right)+\frac{1.9195}{\sqrt{I\left[ M \right]}}$ (3)

where γ is the fraction of GC basepairs.

The effective width^4^ of the DNA is described by Equation 4:

$w=\lambda_{D}\left[ 0.7704+log\left( \frac{\upsilon^{2}}{2\epsilon\epsilon_{0}k_{B}T\kappa} \right) \right]$ (4)

Lee.S et.al^8^  used **Equation 4** to calculate the effective width for a range of ionic strengths, reported in **Supplementary Figure 1A**. From this, a simple log-log plot (**Supplementary Figure 1B**) gives a relation that can be used to extrapolate the effective width for higher ionic strengths. Given these values and equations, the relation between the ionic strength and the extension of DNA under confinement can be plotted to give a theoretical estimation, **Supplementary Figure 1C**.


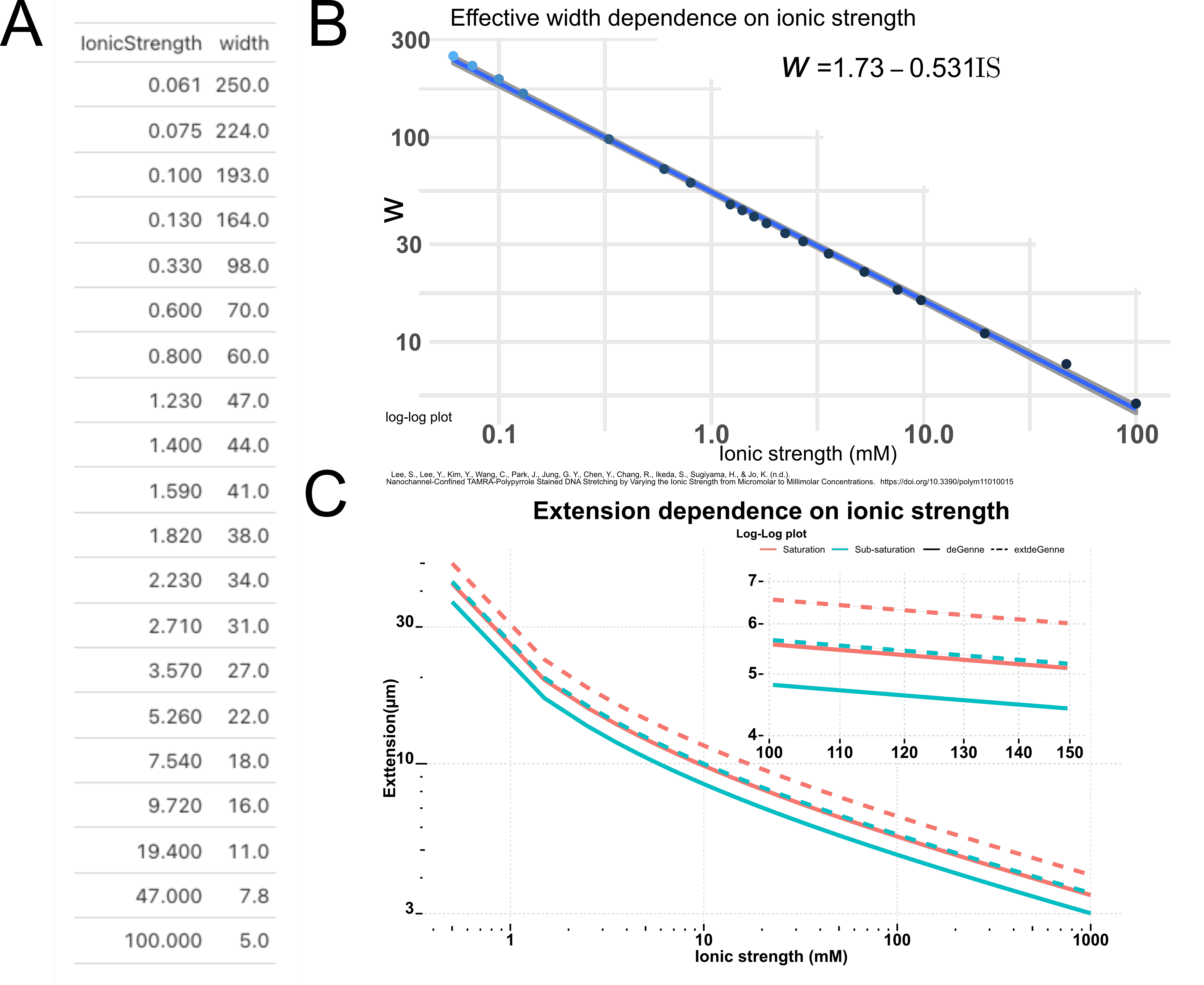


Supplementary Figure 1 **A** Table of ionic strength and the associated width as reported by Lee.S et.al^8^ **B** The effective width dependency on ionic strength and the associated equation that describes the relation. **C** Plot of the theoretical extension of a l-DNA molecule under YOYO-1 saturation and sub-saturation in both the deGenne and extended deGenne regime. The inset highlights the ionic strength range primarily used in this study.

Using the theoretical extension calculated in **Supplementary Figure 1,** the ratio between the observed and theoretical extension was calculated for each experimental condition in **Supplementary Figure 2**.


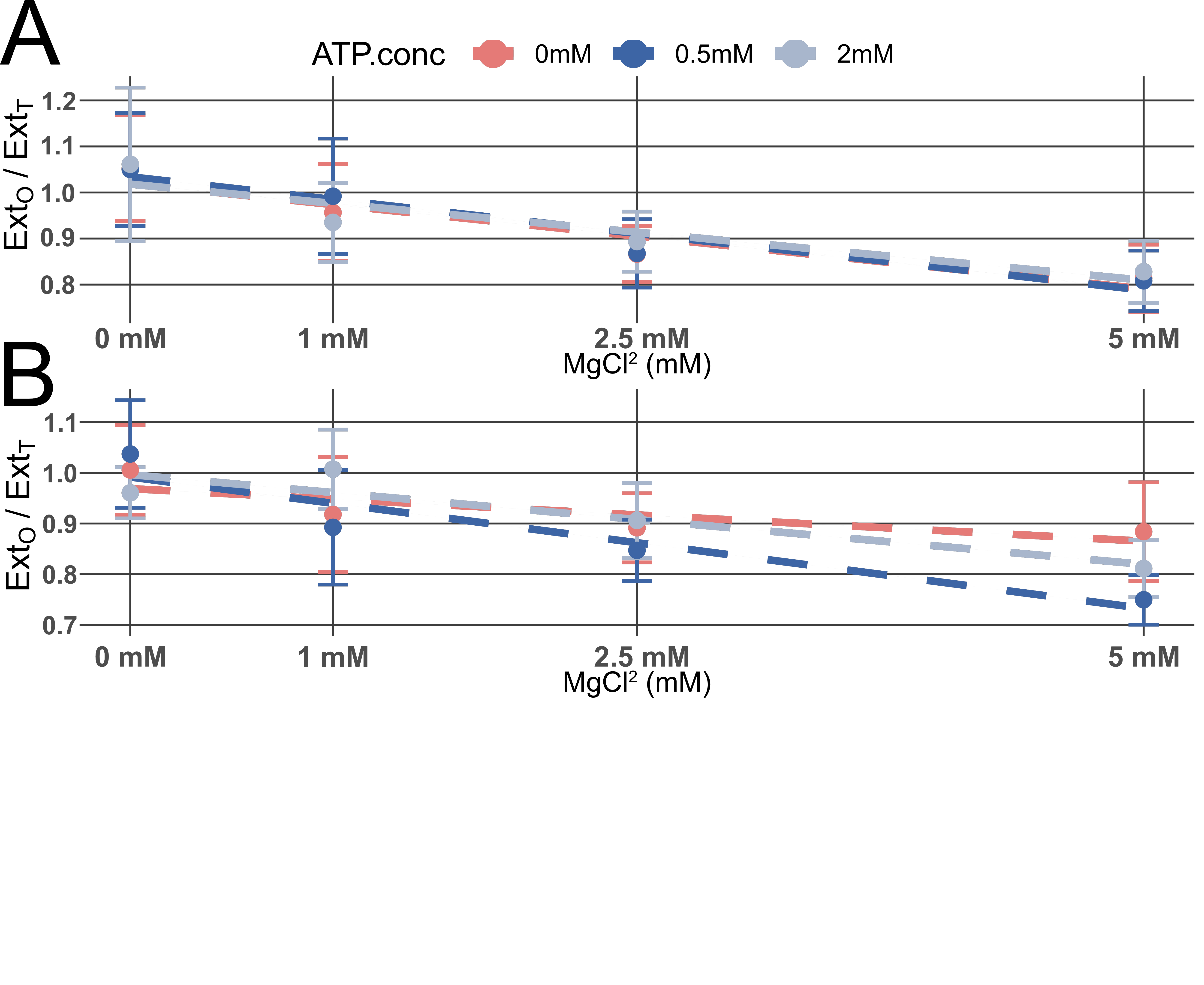


Supplementary Figure 2 Ratio between observed and theoretical extension for varying concentrations of MgCl_2_ and ATP under two different dye:bp ratios. **A**. 1:10 **B**. 1:3

# Single molecule clustering

The monomeric fraction of each dataset was isolated by a two-step clustering method where the first step included a 3D dbscan^9^  clustering using the extension, standard deviation, and intensity as variables. This allowed for the removal of molecules with values far from the core clusters which could be assumed to be outliers or otherwise not representative. The second step involved a simple 1D k-means^10^ clustering to find the most likely threshold between the monomeric and fragment fraction.

# Determination of binding constants

#### McGhee von Hippel

The McGhee and von Hippel model describes the non-cooperative binding of ligands to a DNA lattice^11,12^ and is defined as:

$\frac{\nu}{\left[ L_{f} \right]}=K_{a}(1-n\nu)\left( \frac{1-n\nu}{1-(n-1)\nu} \right)^{n-1}$ (5)

Where [L_f_] is the molar concentration of free ligand, n is the size of the binding site, K_a_ the intrinsic binding constant and $\nu$ the ratio between bound ligand and total concentration of basepairs.

A simplified form of this model was fitted to the data which were derived from the equilibrium constant: $K=\frac{\left[ LS \right]}{\left[ L_{f} \right]\left[ S_{f} \right]}$ and conservation of mass: ${[L}_{T}]={[L}_{f}]+[LS]$ and ${[S}_{T}]={[S}_{f}]+[LS]$ where L is ligand, S is substrate and subscript *f* and *T* is *free* and *Total*. By expressing [LS] in terms of K, substitution gives ${[S}_{T}]={[S}_{f}]+K\left[ L_{f} \right][S_{f}]$. This gives an expression: ${[S}_{f}]=\frac{{[S}_{T}]}{1+K\left[ L_{f} \right]}$ which in turn can be substituted into the expression for [LS] to yield: $[LS]=\frac{K\left[ L_{f} \right]{[S}_{T}]}{1+K\left[ L_{f} \right]}$

This expression is further substituted into the expression for ${[L}_{T}]$, yielding:

${[L}_{T}]={[L}_{f}]+\frac{K\left[ L_{f} \right]{[S}_{T}]}{1+K\left[ L_{f} \right]}$

After adding a term for the total concentration of binding sites the final expression becomes:

${[L}_{T}]={[L}_{f}]+\frac{K\left[ L_{f} \right]}{1+K\left[ L_{f} \right]}\left( \frac{{[S}_{T}]}{n} \right)$ (6)

In the following tables, the binding constant (K), the calculated binding sites (N), the R-squared (R^2^), the residual Sum of Squares (RSS), the total sum of squares (TSS) and the root mean squared error (RMSE) for the different MvH fits from Figure 4 are shown.

R^2^ gives the percentage of variability that is explained by the model, with 1 being a perfect fit and is defined as: $R^{2}=1-\frac{RSS}{TSS}$

RSS is defined as the sum of the squares of the residuals: $RSS=\sum_{i=1}^{n} {(y_{i}-\hat{y}_{i})}^{2}$ with $y_{i}$ being the actual value, $\hat{y}_{i}$ the predicted value and n the number of data points.

The RSS indicates the difference between the actual and predicted values, with a lower RSS meaning a better fit.

TSS is defined as the total variance in the dependent variables: $TSS=\sum_{i=1}^{n} ({y_{i}-\bar{y})}^{2}$ with $\bar{y}$ being the mean of $y_{i}$.

RMSE is an error metric used to measure the average magnitude between the observed and predicted values, with a lower RMSE indicating a better fit: $RMSE=\sqrt{\frac{RSS}{n}}$

Based on the afore-mentioned parameters, the model fit was deemed valid.


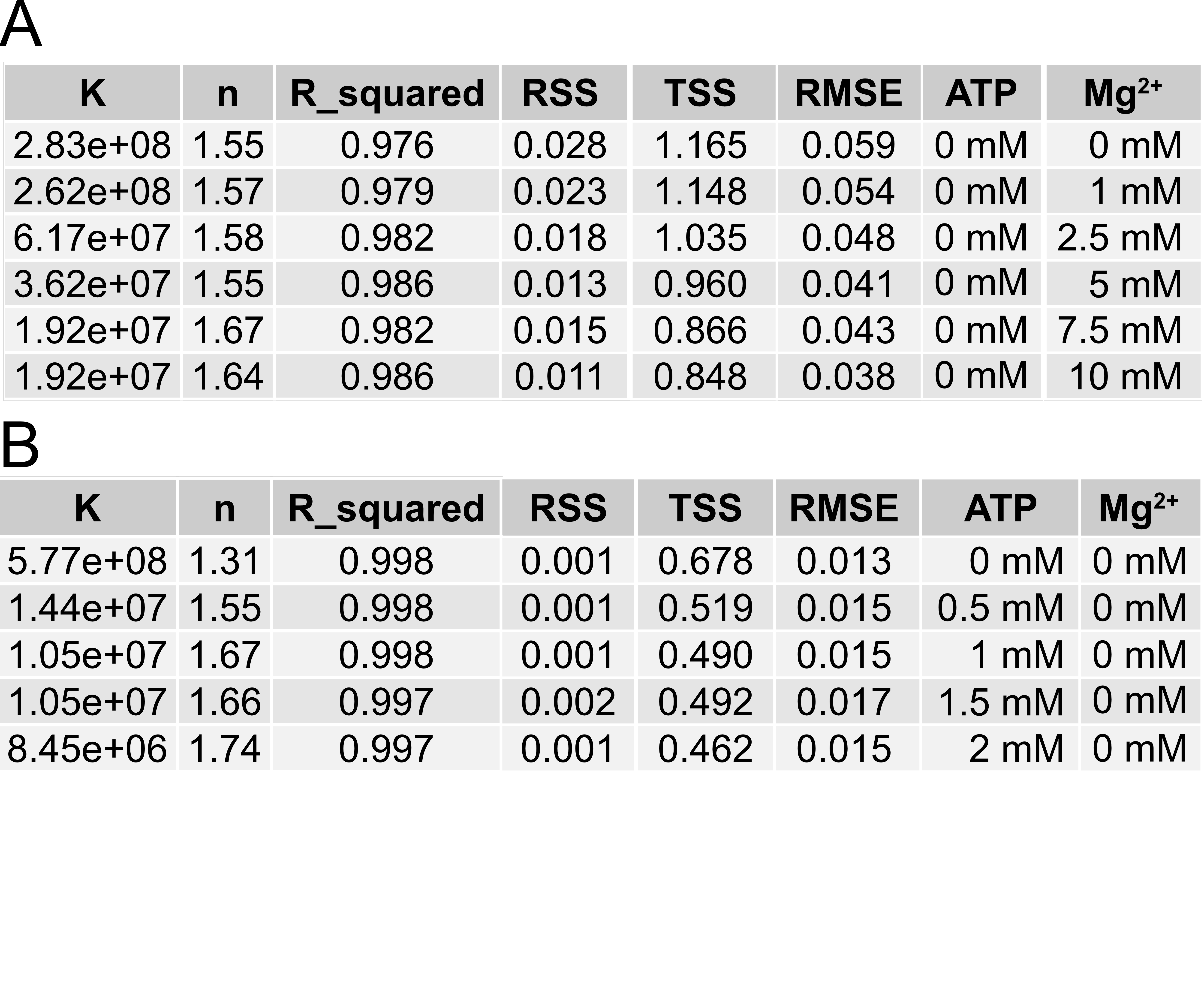


Supplementary Table 1 **A** Table with extracted binding constants and n from fitting of the described MvH model in Equation 6 to fluorescence measurements data from experiments with fixed concentration of YOYO-1, DNA and ATP with varying concentrations of Mg^2+^. The table also reports the R^2^, RSS, TSS, RMSE for each fit. **B** Same as **A** but for experiments with a fixed concentration of Mg^2+^ and varying concentrations of ATP.


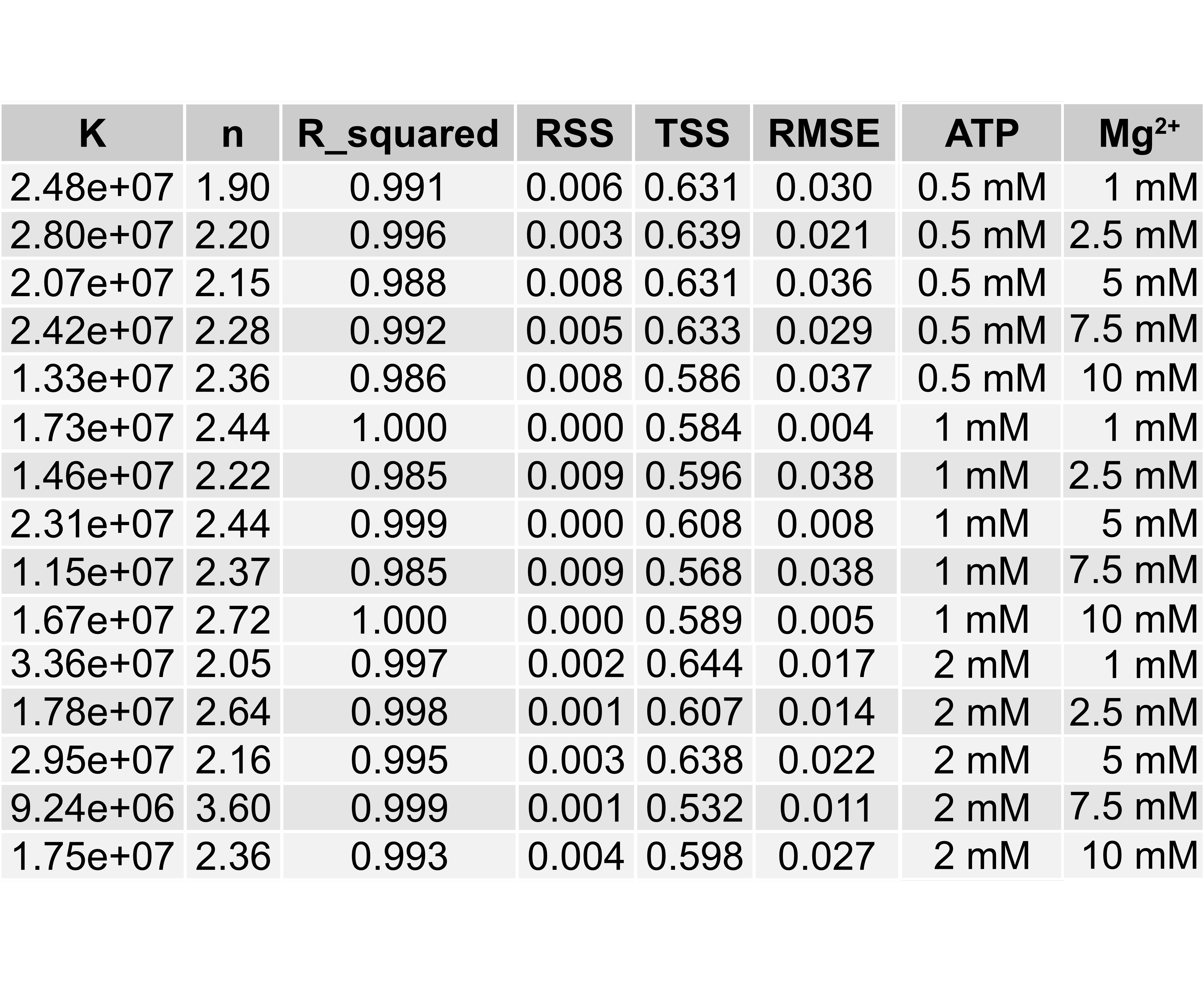


Supplementary Table 2 Table with extracted binding constants and n from fitting of the described MvH model in Equation 6 to fluorescence measurements data from experiments with fixed concentration of YOYO-1 and DNA but with varying concentrations of ATP and Mg^2+^. The table also reports the R^2^, RSS, TSS, RMSE for each fit.

#### Scatchard plot and Hill plot

A scatchard plot was constructed according to^13^ :

$\frac{{[L]}_{total}}{f}=\frac{1}{NK_{f}(1-f)}+\frac{{[M]}_{total}}{N}$ (7)

Where [L]_total_ and [M]_total_ is the YOYO-1 and DNA concentration respectively, N is the total number of binding sites, K_f_ the binding affinity and f the fractional fluorescence. Plotting [L]_total_ / f against 1/(1-f) yields a linear plot with the slope of 1/NK_f_ and intercept [M]_total_/N.

Hill plots were constructed from the Hill equation^14^ :

$\theta=\frac{{[L]}^{n}}{K_{d}+{[L]}^{n}}$ (8)

Where $\theta$ is the fraction of bound ligand, [L] the total ligand concentration, Kd the apparent dissociation constant, and n, the Hill coefficient.

The Hill plot is constructed by rearranging and taking the logarithm which yields:

$\log\left( \frac{\theta}{1-\theta} \right)=n\log([L])-\log\left( K_{d} \right)$ (9)

By plotting $\log\left( \frac{\theta}{1-\theta} \right)$ against $\log([L])$ gives a line with slope n and intercept $\log\left( K_{d} \right)$ from which the binding constant can be readily extracted.


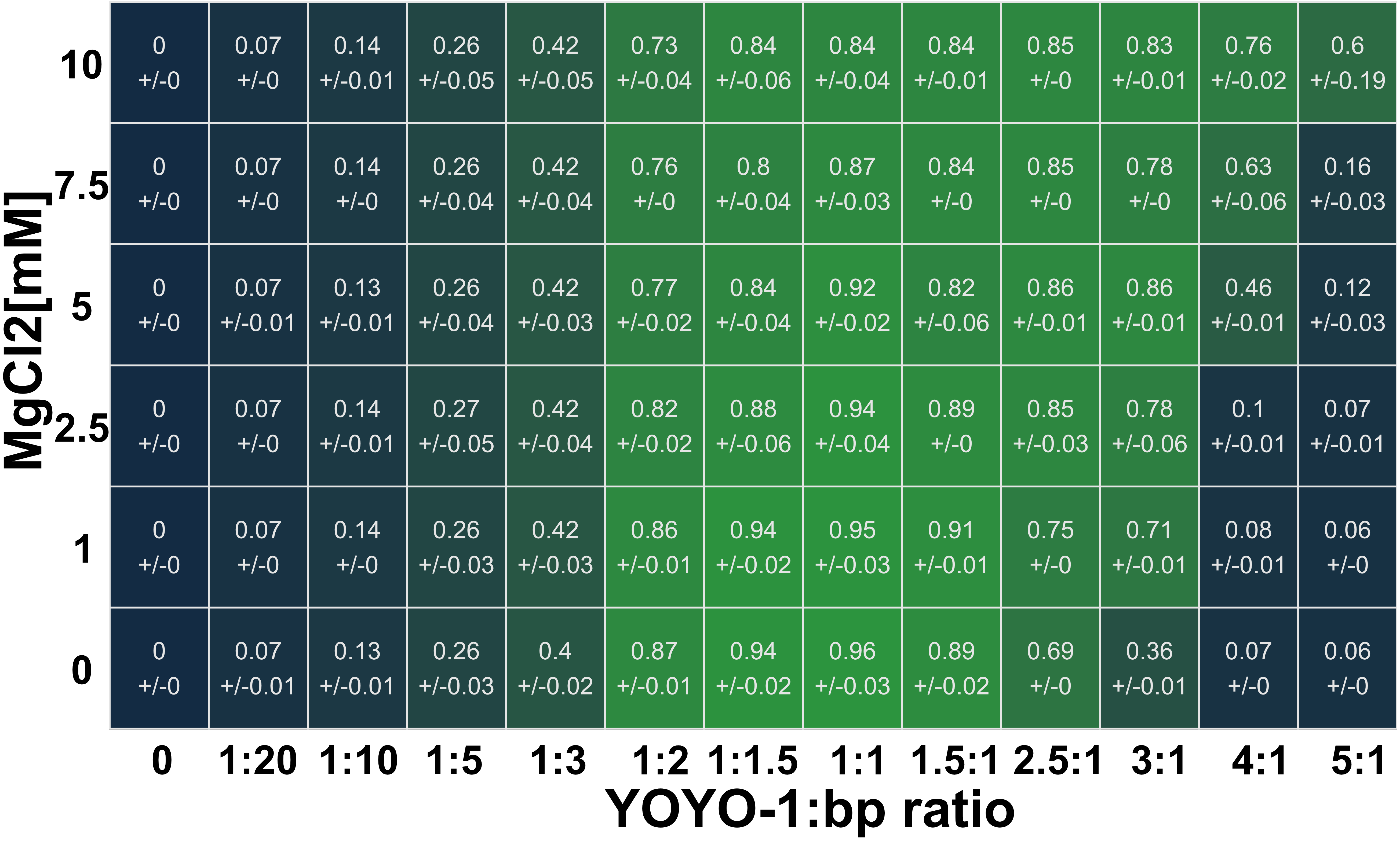


Supplementary Figure 3 Heatmap reporting the recorded fluorescence readings for varying conditions of MgCl_2_ and YOYO-1. The data is normalized and averaged across three replicates^.^


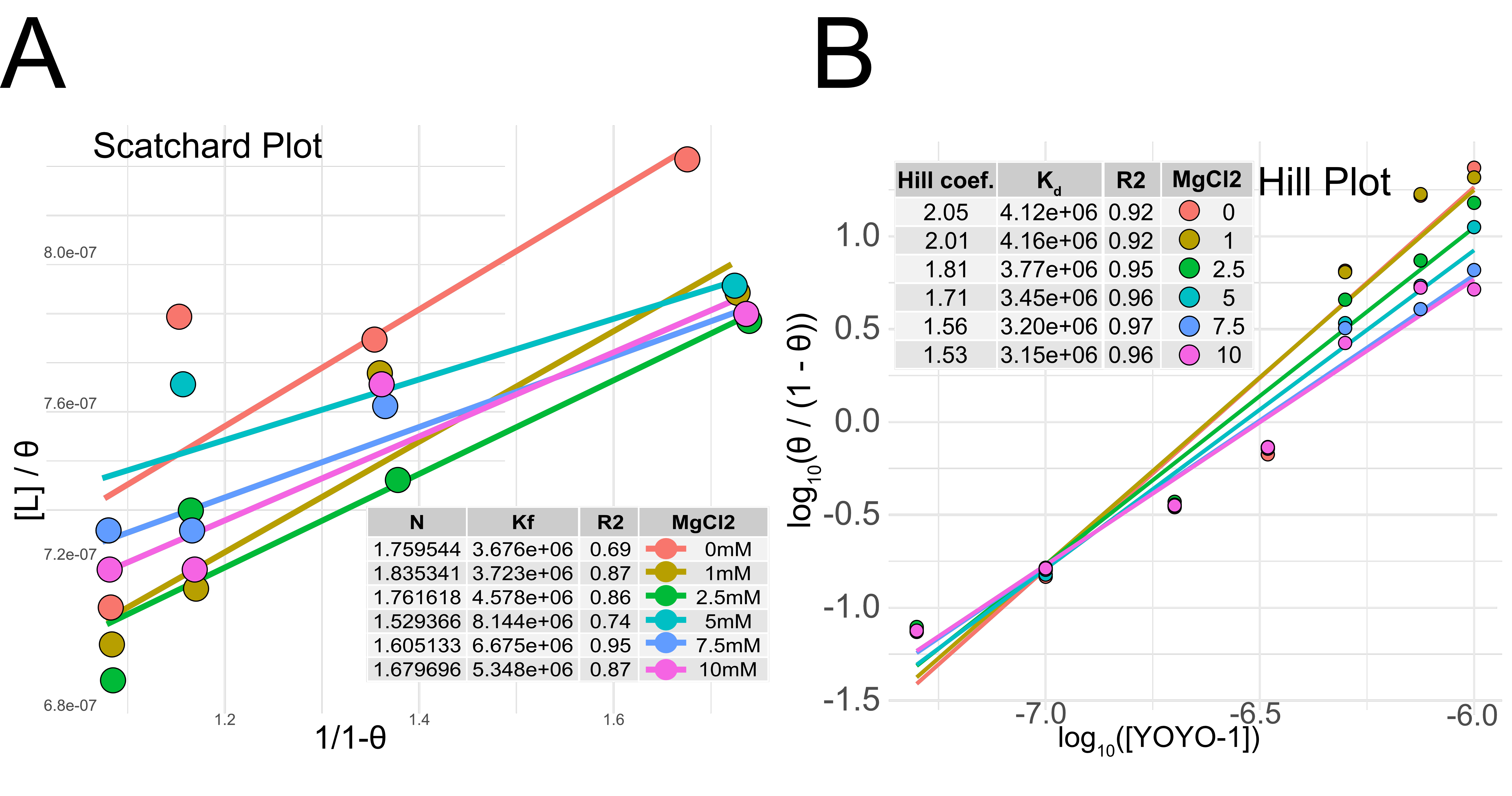


Supplementary Figure 4 **A** Scatchard analysis of fluorescence data from YOYO-1 bound to DNA under variable MgCl_2_ concentrations. The data is truncated to YOYO-1 concentrations below 0.5 µM. The inset table reports the associated binding constant and the binding site size for each MgCl_2_ concentration as well as estimates of the fit. **B** Hill plot with fitted lines and a table with calculated coefficients for all MgCl_2_ levels with YOYO-1 concentrations ranging from 0.05-1 µM (1:20 to 1:1 dye:bp ratios).


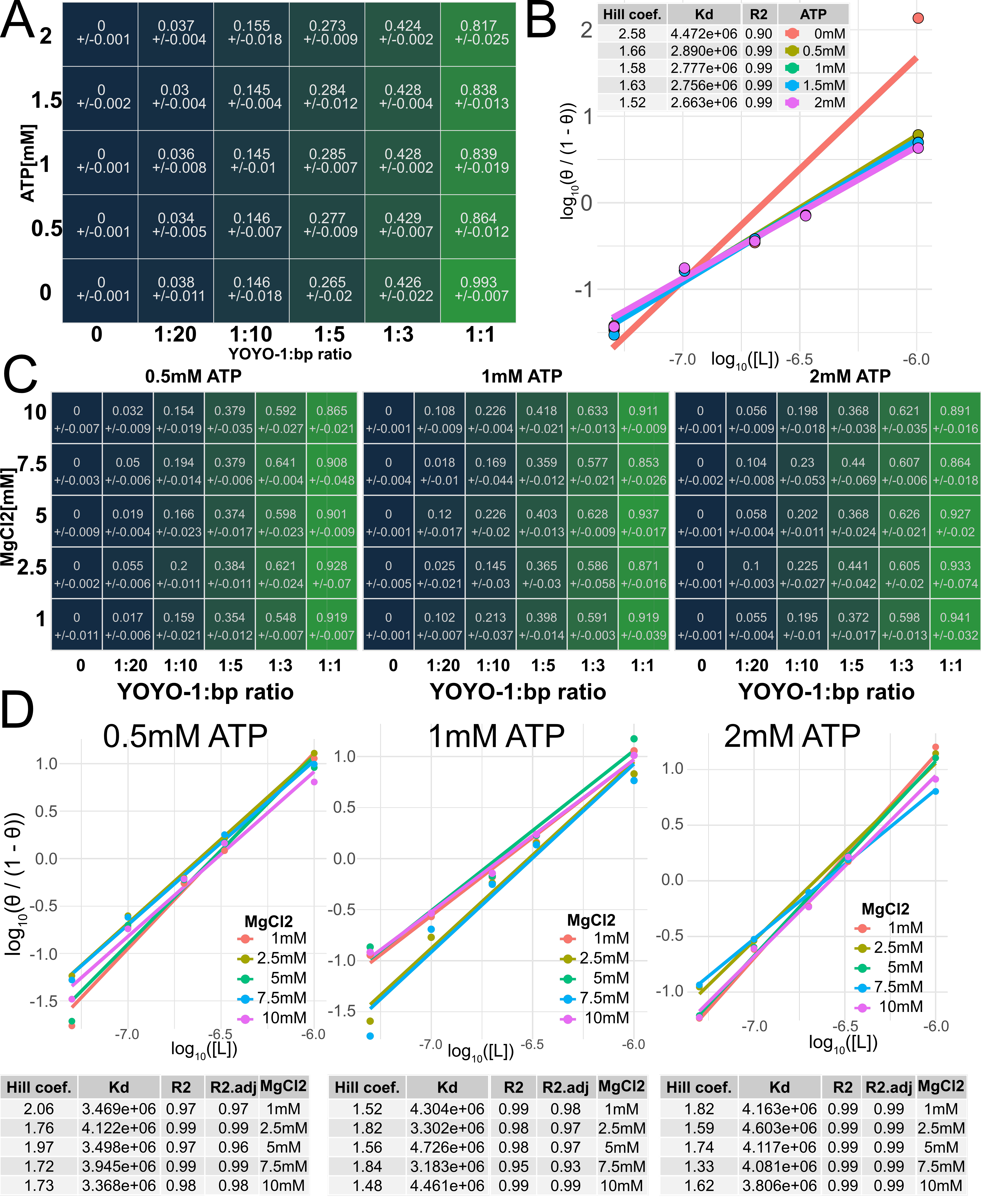


Supplementary Figure 5 **A** Heatmap reporting the recorded fluorescence readings for varying conditions of ATP and YOYO-1. The data is normalized and averaged across three replicates^.^ **B** Hill plot with fitted lines and a table with calculated coefficients for all ATP levels with YOYO-1 concentrations ranging from 0.05-1 µM (1:20 to 1:1 dye:bp ratios). **C** Heatmap reporting the recorded fluorescence readings for varying conditions of MgCl_2_, ATP and YOYO-1. The data is normalized and averaged across three replicates^.^ **D** Hill plots with fitted lines and a table with calculated coefficients for all ATP and MgCl_2 l_evels with YOYO-1 concentrations ranging from 0.05-1 µM (1:20 to 1:1 dye:bp ratios).

# References

1. Team, R. C. R: A Language and Environment for Statistical Computing. (2024).

2. Wickham, H. ggplot2: Elegant Graphics for Data Analysis. (2016).

3. Frykholm, K., Müller, V., Dorfman, K. D. & Westerlund, F. DNA in nanochannels: theory and applications. *Quarterly Reviews of Biophysics* (2022) doi:10.1017/s0033583522000117.

4. Reisner, W., Pedersen, J. N. & Austin, R. H. DNA confinement in nanochannels: Physics and biological applications. *Reports on Progress in Physics* **75**, (2012).

5. Alizadehheidari, M. *et al.* Nanoconfined Circular and Linear DNA: Equilibrium Conformations and Unfolding Kinetics. *Macromolecules* **48**, 871–878 (2015).

6. Werner, E. & Mehlig, B. Confined polymers in the extended de Gennes regime. *PHYSICAL REVIEW E* **90**, 62602 (2014).

7. Gupta, D. *et al.* Experimental Evidence of Weak Excluded Volume Effects for Nanochannel Confined DNA. *ACS Macro Lett.* **4**, 759–763 (2015).

8. Lee, S. *et al.* Nanochannel-Confined TAMRA-Polypyrrole Stained DNA Stretching by Varying the Ionic Strength from Micromolar to Millimolar Concentrations. doi:10.3390/polym11010015.

9. Hahsler, M., Piekenbrock, M. & Doran, D. dbscan : Fast Density-Based Clustering with R. *J. Stat. Softw.* **91**, (2019).

10. Wang, H. & Song, M. Ckmeans.1d.dp: Optimal k-means Clustering in One Dimension by Dynamic Programming. *R J.* **3**, 29 (2011).

11. McGhee, J. D. & Hippel, P. H. von. Theoretical aspects of DNA-protein interactions: Co-operative and non-co-operative binding of large ligands to a one-dimensional homogeneous lattice. *J. Mol. Biol.* **86**, 469–489 (1974).

12. Rocha, M. S. Revisiting the neighbor exclusion model and its applications. *Biopolymers* **93**, 1–7 (2010).

13. Healy, E. F. Quantitative Determination of DNA–Ligand Binding Using Fluorescence Spectroscopy. *J. Chem. Educ.* **84**, 1304 (2007).

14. Stefan, M. I. & Novère, N. L. Cooperative Binding. *PLoS Comput. Biol.* **9**, e1003106 (2013).
